# Laser Evoked Potentials in Early and Presymptomatic Huntington's Disease

**DOI:** 10.1155/2016/8613729

**Published:** 2016-03-21

**Authors:** Marina de Tommaso, Giovanni Franco, Katia Ricci, Anna Montemurno, Vittorio Sciruicchio

**Affiliations:** Apulian Referral Center for Huntington's Disease, Basic Medical Sciences, Neuroscience and Sensory System Department (SMBNOS), University of Bari Aldo Moro, Italy

## Abstract

Pain was rarely studied in Huntington's disease (HD). We presently aimed to extend our previous study on pain pathways functions by laser evoked potentials (LEPs) to a larger cohort of early unmedicated HD patients and a small group of presymptomatic HD (PHD) subjects. Forty-two early HD patients, 10 PHD patients, and 64 controls were submitted to LEPs by right-hand stimulation. Two series of 30 laser stimuli were delivered, and artifact-free responses were averaged. The N1, N2, and P2 latencies were significantly increased and the N2P2 amplitude significantly reduced in HD patients compared to controls. In the HD group, the LEPs abnormalities correlated with functional decline. PHD subjects showed a slight and insignificant increase in LEPs latencies, which was inversely correlated with the possible age of HD clinical onset. Data of the present study seem to suggest that the functional state of nociceptive pathways as assessed by LEPs may be a potential biomarker of disease onset and progression. The assessment of pain symptoms in premanifest and manifest HD may also open a new scenario in terms of subtle disturbances of pain processing, which may have a role in the global burden of the disease.

## 1. Introduction

Huntington's disease (HD) is an inherited autosomal dominant disorder, the phenotypic expression of which consists of invalidating motor, cognitive, and psychiatric symptoms, linked to the progressive dysfunction and neuronal death in corticostriatal circuits [1]. The causative gene (mutated huntingtin, HTT) is inherited in 50% of first-degree relatives, and the genetic test provides for the individuation of presymptomatic subjects. The onset of HD is associated with the first appearance of chorea movements while the possible early cognitive or psychiatric impairment is frequently supposed in clinical practice, before HD diagnosis is done [2]. The CAG replication may predict age at onset [3], but the early stage of neurodegeneration and pathophysiological changes is not evident in clinical practice [4]. The best clinical and instrumental assessment of the presymptomatic stage may provide for a combination of potential biomarkers and improve the knowledge about the neuronal circuits which are affected by mutated HTT even before motor symptoms appear.

Pain is a fundamental function of life, and nociceptive inputs are conducted via specific pathways (the spinothalamic tract) and processed at cortical level by the so-called pain matrix, which includes both cortical areas specifically devoted to pain processing and associative areas integrating salient stimuli for the potential motor response [5]. Increasing interest is growing toward pain expression in different types of dementia [6]. Pain has been also extensively evaluated in extrapyramidal disorders as Parkinson's disease, given that patients report pain symptoms even in the early stage [7], while very few reports focused on the pain in HD patients, despite motor symptoms as dystonia or muscle skeletal damage consequent to postural abnormalities that would cause discomfort. Scherder and Statema [8] described pain in 11 among 19 patients with advanced HD, which had been underestimated and not successfully treated. In a previous study, we examined a cohort of 28 HD patients by means of laser evoked potentials, which are a reliable tool for the detection of pain pathways dysfunctions at both peripheral and central level [9, 10]. In that study, we found prolongation of N2 and P2 cortical waves, which was correlated with disease severity [9]. Slowing of pain processing may interfere with sensory-motor integration [11] with an impact on the general outcome of the disease, even in the early stage. A general impairment in negative emotion recognition including empathy for pain was also found in manifest HD [12], so it is conceivable that the cortical processing of negative stimuli potentially preceding an adversative motor response may be an early phenotypic HD expression.

In the present study, we aimed to confirm the previous laser evoked potentials findings [9], by the evaluation of a new larger cohort of early nonmedicated HD patients and a small sample of genetically predisposed relatives, in order to establish whether the slowing of pain processing may be present in the early and presymptomatic phase of HD.

## 2. Methods

### 2.1. Subjects

Forty-five consecutive nonmedicated HD patients, who came for the first time to our HD regional referral center, were enrolled. Twenty relatives who voluntarily decided to be submitted to the genetic test were examined by means of laser evoked potentials (LEPs). Only the 10 cases that presented with CAG replication ≥39, without current clinical signs of HD onset, were included in the PHD group. The criterion for HD onset was the appearance of chorea movements [2]. Sixty-four healthy volunteers, selected among the hospital staff, were examined. Exclusion criteria were the current use of CNS drugs, the evidence of general medical and other neurological diseases, including present peripheral neuropathies and metabolic diseases as diabetes and chronic renal failure with potential risk for these conditions, a history of HD > 5 years, and a Mini-Mental State Examination score ≤26. We did not exclude patients with chronic lumbar and sacral radiculopathies from spondylarthrosis, which would not interfere with LEPs from hand stimulation. Three HD patients were excluded for severe chorea which disturbed the LEPs recording.

Demographic and clinical data are reported in Table 1. The HD patients were older compared to both PHD and controls. Considering that a linear correlation was present between main LEPs features and age, this was introduced as a covariate in statistical comparisons (see below). For PHD, the presumable age of onset was computed, applying the formula log (age) = *α* + *β* (CAG number repeats), where *α* = 6.16 and *β* = −0.053 [3]. We considered the difference in years between the current age and the presumable age of disease onset, as the expected time of illness onset.

### 2.2. Clinical Evaluation

All patients and PHD patients were submitted to the Mini-Mental State Examination (MMSE) [13] to exclude severe cognitive impairment.

In addition, patients and PHD cases underwent the motor section of Unified Huntington's Disease Rating Scales (UHDRS) [14] and the Total Functional Capacity Scale [15]. The sensory functional status was assessed in HD patients, PHD patients, and controls by clinically standardized evaluation to explore touch, pinprick, pressure, cold, heat, and vibration. To evaluate the presence and characteristic of pain, the short form of Brief Pain Inventory (BFP) [16] was applied to HD and PHD subjects. Chronic pain was assessed according to the IASP (International Association for the Study of Pain) criteria [17]. The Ethical Committee of Bari Policlinico General Hospital approved the study, and each subject signed an informed consent.

### 2.3. CO_2_ Laser Stimulation and LEPs Recording

LEPs were recorded in the Laboratory of Neurophysiopathology of the Pain Unit of our department.

Each subject was seated in a comfortable chair, positioned in a quiet room with an ambient temperature of 21–23°C, in an awake and relaxed state. Subjects and experimenters wore protective goggles during data acquisition. The pain stimulus was a laser pulse (wavelength: 10.6 *μ*m) generated by a CO_2_ laser (Neurolas; Electronic Engineering, Florence, Italy; http://www.elengroup.com/). The location of the impact on the skin was slightly shifted between two successive stimuli, to avoid the sensitization of the nociceptors. The CO_2_ laser stimuli were delivered at fixed 25 ms duration, while intensity was changed in increasing steps of 1,5 Watts in order to individuate the pain threshold, judged by a 10-point verbal analog scale in which “0” corresponds to no sensation, “4” to the pain threshold (painful pinprick), and “10” to intolerable pain. We paid attention to settling the laser power at 1,5 Watts, 1 step above the individual pain threshold in all cases [18], with a VAS value of 5-6 in more than 50% of 20 stimuli. We placed four electrodes at Cz, T3, T4, and Fz positions, with the reference electrode at the nasion; the T3 and T4 electrodes were referred offline to Fz, in order to detect the N1 component [10]. Another electrode was placed above the right eye to record the electrooculogram. Signals were amplified, filtered (0.5–80 Hz), and stored in a biopotential analyzer (Micromed System Plus, Italy). Two series of 30 laser pulses were applied to the dorsum of the right hand, with an interstimulus interval of 10 sec and an interseries interval of at least 5 min. Patients and healthy controls were requested to pay attention to the stimuli. At the end of each stimulation series, all subjects were requested to rate the pain induced on average by the 30 laser stimuli, using a 0–100 visual analog pain scale (laser pain VAS), in which the white color corresponded to 0 (no pain) and intense red to 100 (the most severe pain imaginable). Patients and controls were requested to individuate the number which corresponded to the color expressing the intensity of the perceived laser pain. Although many patients and controls were also submitted to LEPs recording from left-hand stimulation, in other cases, the short time available for examination did not enable completing the two hands, so in the present study, we decided to report only the results from the right-hand stimulation.

All cases included in the study were also submitted to standard electroneurography, in order to exclude peripheral neuropathies. The standard neurophysiological examination was normal in all HD patients, PHD patients, and controls.

### 2.4. LEPs Analysis

An investigator blind to the clinical condition analyzed the LEPs for 1 s, with a 100 ms prestimulus time, at a sampling rate of 512 Hz. All runs containing transient activities that exceeded 65 *μ*V at each recording channel were excluded from the average by an automatic artifact rejection algorithm. In addition, further artifacts were visually inspected and an average of at least 15 artifact-free responses was obtained offline. We performed the baseline correction feature by the subtraction of the DC offset in the 1 sec poststimulus time, according to ASA software, vers. 4.7.3, by ANT neuro (http://www.ant-neuro.com/). For each stimulation site, an average across the two series of stimuli was obtained.

LEPs were identified based on their latency and distribution, and three responses were labeled according to Valeriani et al. [19]. The N2 and P2 components were detected at the vertex (Cz), as a positive-negative complex in the time range 180–450 msec, while the N1 component was checked at T3-Fz, as a smaller negative wave in the latency range 150–250 msec [9, 19]. Absolute latencies of the scalp potentials were measured at the highest peak of each response component. The amplitude of the N1 was measured from the baseline while the peak-to-peak amplitude was considered for the N2/P2 complex.

### 2.5. Statistical Analysis

One-way ANOVA with diagnosis (HD versus PHD versus N) as factor and main LEPs features as variables was performed. In control subjects, there was a linear correlation between age and main LEPs features (linear regression test for N2P2 amplitude *F* = 24.56, *p* < 0.0001; N1 amplitude *F* = 7.58, *p* = 0.008; N1 latency *F* = 4, *p* = 0.049; N2 latency *F* = 4.92, *p* = 0.03, P2 latency *F* = 5.69, *p* = 0.02) so age was included as covariate in ANOVA analysis.

The post hoc Bonferroni test was also employed among groups. In HD group, the correlation between LEPs values and main clinical features was done by means of Pearson correlation test. In PHD, the expected time of illness onset, as well as the UHDRS motor section, was also correlated with LEPs latencies and amplitudes by the partial correlation test, subtracting the age effect. The SPSS vers. 21 was used.

## 3. Results

### 3.1. Clinical Examination

Main clinical features of patients and presymptomatic HD subjects are reported in Table 1. In 6 out of 10 pre-HD patients, few nonspecific motor abnormalities, as slight oculomotor slowing, were present. The expected time of illness onset varied from 1 to 21 years. The MMSE was normal in all PHD cases. Only 3 HD patients reported chronic pain, 2 of mixed nociceptive neuropathic type (low back pain with lumbar radiculopathy from spondyloarthosis) and 1 HD patient, female, who suffered from diffuse muscle skeletal pain (fibromyalgia). No PHD subject suffered from chronic pain. Considering the small number of patients complaining of pain, the BPI items were not reported.

### 3.2. Laser Evoked Potentials

The LEPs features, including pain threshold and VAS values, are summarized in Table 2.

**Figure 1:**
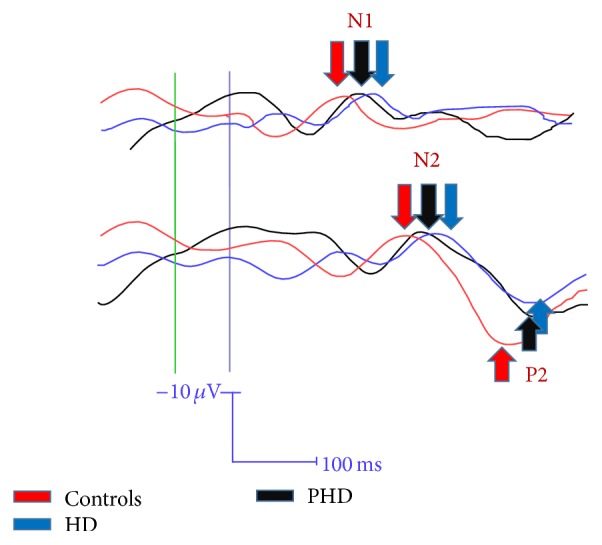
Grand average of N2P2 vertex complex computed in normal subjects (N) (64), HD (Huntington's disease) patients (43), and PHD (presymptomatic HD) subjects (10). The N2 and P2 components are indicated with colored arrows.

**Figure 2:**
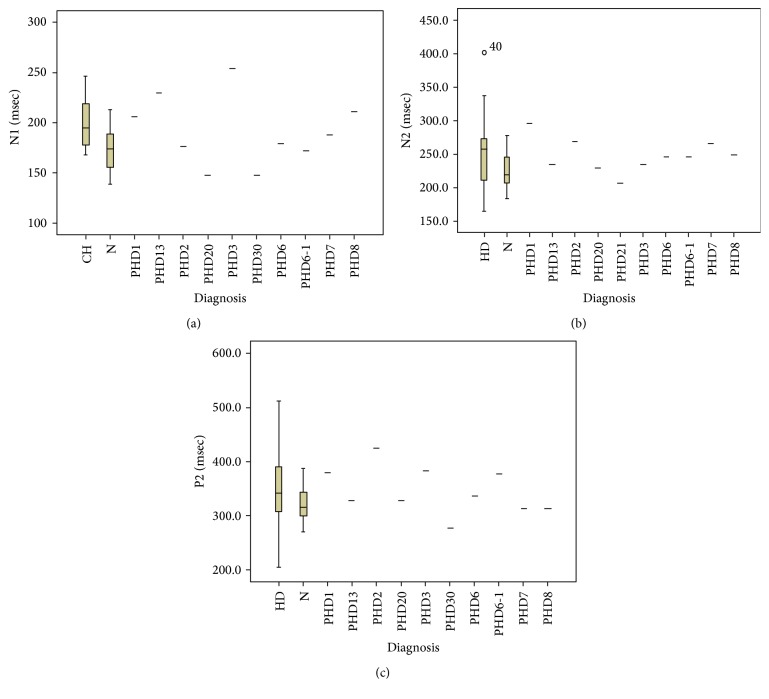
The N1 (a), N2 (b), and P2 (c) latency values are shown for single PHD (presymptomatic HD) cases and N (normal) and HD groups (95% CI). The values are corrected for age. The numbers following the PHD title expressed the expected time of illness onset (in years). Only the PHD21 case, who was an 18-year-old girl, showed values in the lower normal limits. The cases PHD1, PHD2, and PHD3 with a risk for manifest chorea within little time presented with P2 latency in the upper normal limits. The case PHD1 showed significant prolongation of N2 latency. The case PHD2 had significant prolongation of N1 latency.

HD patients presented with significant prolongation of LEPs components latencies and a reduction of N2P2 vertex complex amplitude, in respect to controls (Table 2). A slight LEPs latency increase was present in PHD subjects, where values were in an intermediate range between patients and controls, so they did not differ either from patients or from controls, as shown by the Bonferroni test results (Table 2, Figure 1). The N2P2 amplitude was similar to controls, but not significantly different from HD group. The analysis of single PHD cases showed that subjects who were hypothetically near to the clinical onset of the disease had N2 and P2 latencies in the upper limit of normality (Figures 2(a), 2(b), and 2(c)).

### 3.3. Correlations between LEPs and Clinical Features

In HD patients, the P2 latency was negatively correlated with functional capacities (Pearson correlation: −0.434, *p* < 0.01), expressed by the Total Functional Capacity score; the N2 and N1 latencies were also negatively correlated with the TFC score (N2: −0.327, *p* < 0.05; N1: −0.321, *p* < 0.05); the N2P2 amplitude was positively correlated with the TFC (0.338, *p* < 0.05).

In PHD, the expected time of illness onset was negatively correlated with motor abnormalities and N1, N2, and P2 latencies. The UHDRS motor section was not correlated with the LEPs features (Table 3).

## 4. Discussion

In this study, we confirmed LEPs abnormalities in early HD. In fact, the increased LEPs latencies previously observed in a smaller cohort [9] were confirmed in the present HD group. A significant N2P2 amplitude reduction emerged in the present HD cohort. The consistency of actual results was based on the increased number of HD patients and the exclusion of confounding factors, as the use of centrally acting drugs. The amplitude reduction did not involve the early N1, possibly because this wave is smaller and more variable than the vertex complex [10]. However, both the early temporal and the late vertex components were affected by latency increase, which could suggest A-delta fibers dysfunction at the peripheral level. Given that we carefully avoided including patients affected by peripheral neuropathies and that standard electroneurography examination was normal in all cases, central delay in nociceptive inputs processing may be rather supposed, though the exact mechanism by which the genetic abnormality subtending HD may affect noxious stimuli processing is presently unknown. Although no LEP component may be generated from the basal ganglia [20], these receive all types of somatosensory information in order to modulate nociceptive cortex and organize the possible motor response against potentially dangerous events [21, 22]. Slowing of cortical response to nociceptive stimuli may interfere with sensory-motor integration and affect voluntary motor planning [11]. This phenomenon seems to involve either the early cortical functions of stimulus detection and discrimination, expressed by the N1 wave, or the late vertex N2P2 response induced by the attention and arousal toward relevant stimuli, worthy of possible motor reaction [23, 24]. The impairment of painful stimuli transmission may also cause the cortical degeneration responsible for the LEPs amplitude reduction observed in our HD patients. This abnormal functioning of nociceptive transmission may impair pain feeling and expression, possibly explaining the low number of patients complaining of chronic pain in our HD group, though this finding is not conclusive and needs to be confirmed in larger and normal population controlled studies. The expression of pain is currently a challenge for the management of dementia and neurodegenerative disorders [6]. A possible disturbance in pain symptoms expression was rarely reported in HD [8], with the possibility that even visceral pain would be underestimated and not appropriately treated [25]. In accord with previous results, we did not observe sensory disturbances in our patients [9]. Besides, the laser pain threshold and perception were within normal limits, suggesting that the LEPs abnormalities we observed were not associated with an evident sensory deficit. Despite this, we confirmed that LEPs abnormalities were correlated with impairment in functional capacities, in accord with a previous study [9], while no correlation was found with motor impairment or chorea. The slowing in nociceptive inputs processing may not be a consequence of motor disturbances, but an independent phenomenon which may negatively influence sensory-motor integration, motor planning, and ability in daily living. Accordingly, a progressive reduction of cortical somatosensory evoked potentials in parallel with functional impairment evolution was observed in longitudinal studies of HD patients [26]. Moreover, the deterioration of pain transmission may evolve with the progression of neurodegeneration and the global worsening of the disease, being a phenotypical manifestation of the genetically induced brain changes. In our HD series, the lack of correlation between LEPs abnormalities and illness duration may be explained by the scarce reliability of this feature in marking the real beginning of the disease, with functional capacities being a more consistent sign of disease progression. The slowing in nociceptive inputs processing may be supported by a general impairment in somatosensory or even cognitive functions. However, most of the studies on somatosensory evoked potentials in HD reported a progressive amplitude reduction rather than latency prolongation even in the early phase [26]. Cognitive event-related potentials, as P300, did not show clear abnormalities in early HD [27]. In addition, our patients were not affected by relevant cognitive decline. Although our study lacks control sessions including the not nociceptive somatosensory system and cognitive event-related responses (which were avoided for the long and exhausting procedure), data from other studies on these neurophysiological examinations seem to support the hypothesis that in the early HD the slowing of pain pathways may precede other systems' dysfunction. In regard to presymptomatic HD, very few subjects in PHD state were examined, though the present results seem worthy of discussion and further confirmation in enlarged groups. Based on actual age and CAG expansion [3], our presymptomatic subjects were different in regard to the possible age of clinical onset. Slight motor symptoms as oculomotor disturbances were observed in few HD relatives, being not so relevant to be attributed to HD onset [2]. This slight motor impairment was correlated with risk age, which confirmed that the genetically induced pathological process and huntingtin abnormal functions may start before chorea appearance [2]. In PHD subjects, LEPs latencies were not significantly different either from normal controls or from HD patients. Although the statistical analysis could be affected by the small number of PHD subjects, single cases possibly approaching the clinical diagnosis of HD presented with prolongation of all LEPs waves, which negatively correlated with the supposed time before clinical diagnosis. Early brain degeneration starting before chorea appearance may negatively influence the processing of painful stimuli [28]. The phenotypic expression of presymptomatic HD is useful to apply the potential neuroprotective therapies, so many studies focused on the biomarkers of neurodegeneration [29]. In our small presymptomatic cohort, LEPs latencies seemed normal in younger subjects, so the slowing in painful stimuli processing may be considered a symptom of the stage immediately preceding the clinical diagnosis of HD. This is also confirmed by the clear LEPs abnormalities observed in the early HD patients. The neurophysiological abnormalities we observed would be caused by structural changes in the presymptomatic brain, which seem to predict HD clinical onset [2]. How LEPs abnormalities may be correlated with specific clinical symptoms is actually unclear. Moreover, the possible lower frequency of chronic pain syndromes in pre- and manifest HD people seems worthy of extensive evaluation and comparison to general population. This question would have relevance in the HD management, even in a very early stage, as the impairment in nociceptive stimuli processing seems to be associated with reduced functional capacities. LEPs are a reliable tool to assess the functional state of nociceptive pathways, but they do not always reflect subjective pain perception [10], because compensatory phenomena may occur in the early damaged brain [29] and mask subtle sensory changes. Moreover, LEPs abnormalities may concur with a slight attention deficit possibly present in the presymptomatic HD carriers. There is actually little evidence of cognitive decline before HD clinical appearance [2, 30], and, in addition, cognitive factors influence LEPs amplitudes more than latencies [31]. However, we did not perform a careful cognitive evaluation in our HD and PHD series, given that the MMSE provided only for the exclusion of severe dementia, so the possible correlation between LEPs abnormalities and subtle cognitive impairment deserves further studies.

The ability to recognize others' negative emotions and especially disgust facial expression is impaired in early HD and in the presymptomatic phase [32], so a deficit in processing negative stimuli may be caused by HD pathological changes. However, in a recent study by Baez et al. [12], empathy for pain, which is processed in some cortical areas involved in LEPs generation, as the insula and anterior cingulate [23, 33], was normal in HD relatives and compromised in patients with manifest disease, though some behavioral and cognitive responses may be preserved even in the presence of subtle anatomical and functional brain changes [29].

Other neurophysiological patterns as event-related cognitive potentials (Mismatch Negativity and P300) showed abnormalities in presymptomatic subjects [34]. Surprisingly, the same authors did not confirm the same abnormalities in patients with manifest HD, attributing this apparent puzzling result to possible compensatory upgrading of excitatory circuits occurring in the course of neurodegeneration [34]. In this sense, LEPs abnormal pattern seems to be a more robust indicator of pathological changes progressively occurring in HD brain. 

*Main Study Limitations*. The limited number of presymptomatic HD patients and the lack of a prospective design are major flaws of the present study, together with the absence of a morphometric assessment of cortical areas possibly generating LEPs and of a complete clinical examination, including cognitive and emotional aspects. However, data in manifest HD seem to confirm that LEPs abnormalities may be a feature of early preclinical phase, marking the progression of HD severity and disability.

## 5. Conclusions

Data of the present study seem to suggest that the functional state of nociceptive pathways as assessed by LEPs may be a potential biomarker of disease onset and progression. This is not surprising, given the importance of pain in human life and the influence of basal ganglia on cortical areas devoted to nociceptive stimuli processing. As a matter of fact, this study may indicate the opportunity of more extensive and possibly longitudinal LEPs studies, integrating further clinical and anatomical examination. The assessment of pain symptoms in premanifest and manifest HD may also open a new scenario in terms of subtle disturbances of pain processing, which may have a role in the global burden of the disease.

## Figures and Tables

**Table 1:** Demographic and clinical data in normal subjects (N), Huntington's disease (HD) patients, and presymptomatic Huntington's disease (PHD) patients. The ANOVA analysis shows that age was different among groups. TFC score: total functional capacity score; UHDRS: Unified Huntington's Disease Rating Scale; MMSE: Mini-Mental State Examination. The CAG range is reported. For time from or before illness onset, the range is reported in parentheses. In PHD, negative values indicate the supposed years before clinical HD diagnosis.

	Age	Sex	CAG	Illness onset (years)	UHDRS motor section	TFC score	MMSE	Chronic pain (number)
N	42 ± 16.35	34 F 30 M						

HD	54 ± 11.50	20 F 22 M	39–56	3.23 ± 2.11 (1,5)	32.93 ± 18.97	8.92 ± 3.33	27.1 ± 1.8	3 (mixed pain (2) and fibromyalgia syndrome (1))

PHD	36.62 ± 8.61	5 F 5 M	39–51	−12.20 ± 9.6 (−1, −21)	4.1 ± 4.33	13 ± 0	29.9 ± 0.31	0

	ANOVA *F* = 11.60 *p* < 0.001	Chi square: 0.99 ns						

**Table 2:** Laser evoked potentials features, including laser pain threshold and subjective perception (expressed by visual analog scale (VAS) from 0 to 100) in Huntington's disease (HD) subjects, presymptomatic Huntington's disease (PHD) subjects, and normal controls (N). The results of one-way ANOVA and post hoc Bonferroni test are reported. Significant results are reported in bold font.

Diagnosis	Pain threshold (Watt)	VAS	N1 (msec)	N1 (*μ*V)	N2 (msec)	P2 (msec)	N2P2 (*μ*V)
HD: 43							
Mean	13.5	45	202.45	3.77	250.628	351.69	8.74
SD	5.5	22.2	32.241	2.31	47.76	66.73	6.06
N: 64							
Mean	13.1	43.05	171.00	4.68	227.82	320.13	17.26
SD	4.8	24.46	31.128	3.12	23.68	27.06	11.68
PHD: 10							
Mean	12.9	41.3	185.30	4.20	241	346.1	14.22
SD	5.6	19.14	26.403	3.85	32.9	42.77	11.82
ANOVA (age as covariate)							
*F*		0.98	7.16	1.73	5.52	5.63	5.83
DF		2	2	2	2	2	2
*p*		ns	**0.001**	ns	**0.005**	**0.005**	**0.004**
Bonferroni							
N versus HD patients		ns	**0.001**	ns	**0.004**	**0.004**	**0.003**
N versus PHD patients		ns	ns	ns	ns	ns	ns
HD patients versus PHD patients		ns	ns	ns	ns	ns	ns

**Table 3:** Partial correlation test between laser evoked potentials latencies and amplitudes and expected time of illness onset in 10 presymptomatic HD (PHD) cases (age effect was subtracted). UHDRS: Unified Huntington's Disease Rating Scale.

		UHDRS motor section	N2	P2	N1	N2P2
PHD	10 (DF: 7)	−0.714	−0.723	−0.732	−0.636	0.412
		*p* < 0.05	*p* < 0.05	*p* < 0.05	*p* < 0.05	ns
